# Computational modelling of micro-seizures and focal seizure onset

**DOI:** 10.1186/1471-2202-14-S1-P14

**Published:** 2013-07-08

**Authors:** Yujiang Wang, Peter N Taylor, Gerold Baier

**Affiliations:** 1 Manchester Interdisciplinary Biocentre, 131 Princess Street, Manchester M1 7DN, UK; 2 School of Electrical & Electronic Engineering, Nanyang Technological University, Singapore; 3 Division of Bioscience, Faculty of Life Sciences, University College London, London WC1E 6BT, UK

## 

Pathological micro-domains have been proposed to underpin the generation of local pathological activity, as seen in focal seizures in the epileptic cortex [1-3]. Specifically, so-called micro-seizures have been suggested to be markers for these micro-domains [2,3]. Astonishingly, micro-seizures have also been observed in non-epileptic control patients [3]. This suggests that local activity, such as micro-seizures, only become pathological when in a specific arrangement.

We hypothesize that pathological dynamics could be due to an increased density of micro-domains. To test this, we introduce a computational model on the mesoscopic scale of a 5 × 5 mm^2 ^cortical sheet [4]. The units are modelled as excitable minicolumns. This model also incorporates realistic connectivity schemes observed at this spatial scale [5].

**Figure 1:**
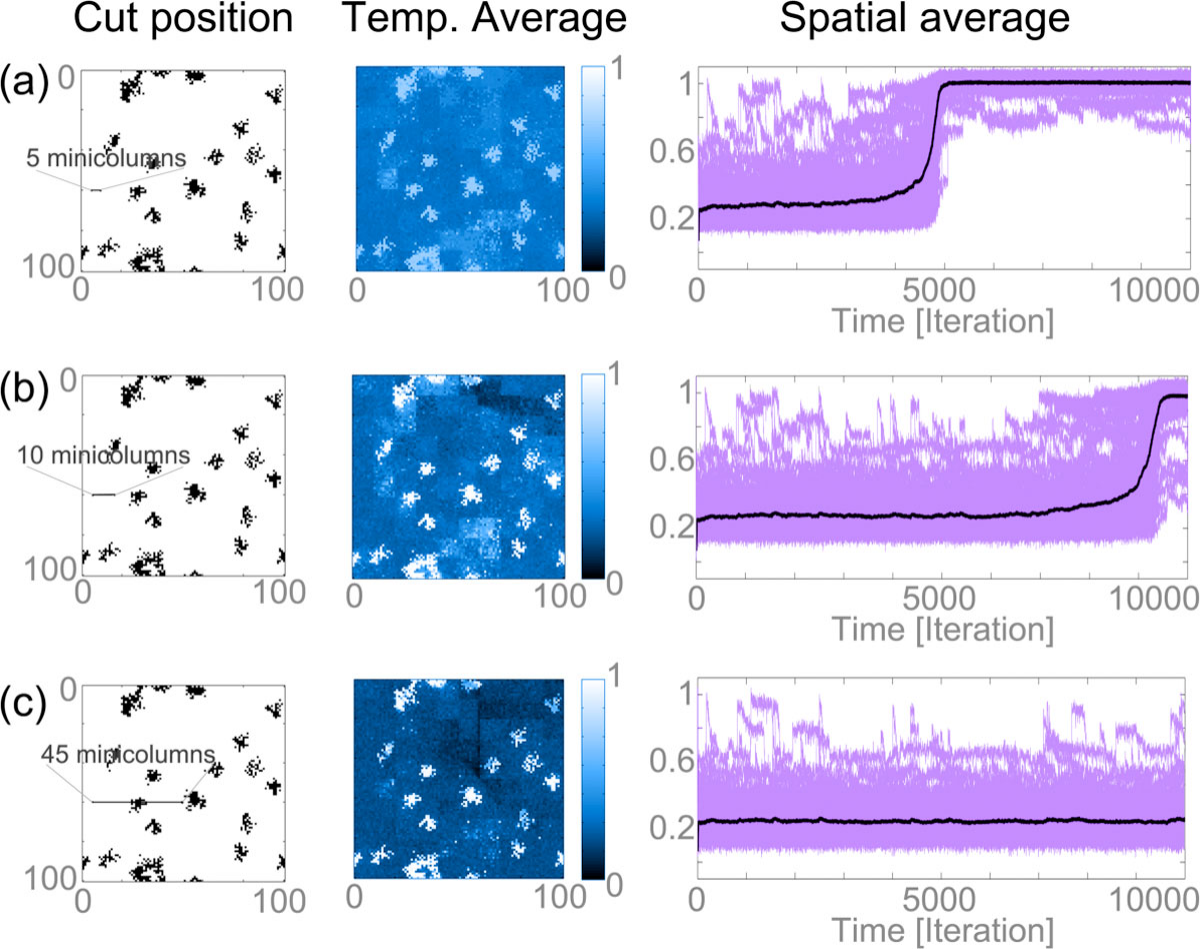
**First column indicated the location of the pathological clusters in black and the position of the micro-incision using the labeled line**. The second column shows the temporal average activity in 10000 simulation steps. The third column indicates the spatial average of the same simulation using the black line and the purple lines show the spatial average of 10 × 10 subsquares, which represent macro-columns in the model. (A) Resulting simulation after a 5 minicolumn long micro-incision. (B) Resulting simulation after a 10 minicolumn long micro-incision. (C) Resulting simulation after a 45 minicolumn long micro-incision.

The model shows occasional, non-pathological micro-seizure occurrences, as well as recruitment of normal tissue into full-blown seizure activity in the presence of dense clusters of hyperactive micro-seizure domains. A specific prediction of this model is that the transition to full-blown seizures can be prevented by using micro-incisions to separate the clusters of abnormally active micro-domains (Figure. 1) [6].
